# Differences in income-related inequality and horizontal inequity in ambulatory care use between rural and non-rural areas: using the 1998-2001 U.S. National Health Interview Survey data

**DOI:** 10.1186/1475-9276-9-17

**Published:** 2010-07-02

**Authors:** Hosung Shin, Jinsook Kim

**Affiliations:** 1 Korea Institute for Health and Social Affairs, San 42-14 Bulkwang-dong Eunpyung-gu, Seoul 122-040, Republic of Korea; 2 Public Health and Health Education Programs, School of Nursing and Health Studies, Northern Illinois University, Wirtz 254, DeKalb, IL 60115, USA

## Abstract

**Background:**

To better understand income-related inequalities in health care use, it is imperative to identify sources of inequalities and assess the extent to which health care use is still related to income after differences in need across the income distribution are accounted for. Little is known regarding rural-urban differences in income-related inequalities and subgroup variation in horizontal inequities in health care use. This study decomposes income-related inequalities in ambulatory care use into contributions of need and non-need factors and compares horizontal inequities of subgroups in rural and non-rural areas.

**Methods:**

This analysis used non-elderly adult samples from the 1998 to 2001 U.S. National Health Interview Survey data. The area of residence was categorized as rural for non-Metropolitan Statistical Area (MSA) and non-rural for MSA. Concentration indices of ambulatory care use were used to gauge income-related inequalities and decomposed into contributing factors. Horizontal inequities were measured using two methods and the results were compared.

**Results:**

Ambulatory care use was disproportionately concentrated in the poor before need adjustment. However, the results of decomposition and horizontal inequity analyses indicate that the pro-poor concentration of health care use was due to greater health care need in low-income groups. Adjusting for need, ambulatory care use was distributed favoring the better-off, to a larger degree in non-rural areas. Health-related variables were the major contributors to income-related inequalities. Non-need factors, including socioeconomic factors, health insurance, and usual source of care, also contributed to income-related inequalities. There were variation in determinants' contributions to income-related inequalities between rural and non-rural populations and subgroup differences in horizontal inequities. Horizontal inequities were greater within non-whites, high school graduates, individuals with private health insurance, and those without a usual source of care with some geographic variation.

**Conclusions:**

Our analysis shows that seemingly pro-poor income-related inequalities in ambulatory care use were largely due to greater health care need among low-income groups. The results demonstrate different contributions of determinants to income-related inequalities and variation in horizontal inequities by subgroup and locale. The findings of this study should help identify targets for policy intervention for each rural and non-rural area.

## Background

Income inequalities have been associated with differences in health outcomes including mortality, mental and physical health, and reproductive outcomes [1-5]. Research suggests two main pathways through which income inequality affects population health: underinvestment in highly inequitable communities and psychological impacts of income inequality on disadvantaged individuals [6,7]. Political units in highly inequitable communities are less likely to invest in infrastructure needed for population health, and members of those communities experience negative health consequences due to psychological factors such as low levels of social cohesion and perception of unfairness [6]. Despite some disagreement on the association [8-10], research continues to show a significant association between income inequality and health disparities in the U.S. [1,2,7,11-13].

Health inequalities are partly attributed to unequal health care use across sociodemographic groups [14,15]. Studies suggest that health care use, especially primary health care use, may ameliorate the negative consequences of income inequality for health [16,17]. Various individual-level factors including health care need, demographic characteristics, socioeconomic status (SES), and health care system factors are likely to contribute to income-related inequalities in health care use [17,18].

Horizontal equity is a widely accepted concept in health inequality research [12,19]. The horizontal equity principle calls for equal treatment of people in equal need regardless of sociodemographic factors such as income, education, place of residence, and race [20]. Since variation in health care use due to differences in health status is unavoidable (i.e., sick people using more health care than healthy people), income-related inequality itself is not considered inequity in health care use [21]. Therefore, to measure inequity correctly, health care need of different groups should be accounted for. Horizontal inequity in health care use measures the degree to which health care use is related to income after controlling for differences in need across the income distribution [22,23].

Horizontal inequity index (*HI*_*wv*_), a measure of inequity in health care use, can be obtained either (1) by calculating the concentration index of need-standardized health care use or (2) by subtracting the concentration index of need-predicted (or need-expected) use (*C*_*N*_) from that of actual care use (*C*_*U*_) [21-24]. Since the distribution of dependent variables (e.g., number of physician visits) in health care use models typically does not follow the normal distribution, non-linear regression models such as logistic and truncated and generalized negative binomial regression models, instead of linear regression models, are used in computing health care use-related indices [21]. Due to the linear approximation steps involved in calculating concentration indices based on non-linear models, the horizontal inequity measured by the concentration index of need-standardized care use is not identical to the index obtained by subtracting the concentration index of need-predicted use from the concentration index of actual care use [24].

A negative value of a concentration index signifies health care need/use favoring lower-income groups. When the concentration index is positive, health care need/use is more concentrated in higher-income groups. If the concentration index is zero, health care need/use is distributed equally regardless of income level [25]. A positive *HI*_*wv *_indicates a higher share of health care use of higher-income groups than their share of need, indicating horizontal inequities favoring the better-off. On the contrary, a negative value of *HI*_*wv *_represents horizontal inequities favoring the poor given their share of need [19].

When individual-level information is available from microdata (e.g., survey data), a horizontal inequity index can be obtained based on the estimates from indirect need standardization, in which non-need variables in addition to need variables are included in regression [21,24]. Here, the concentration index of need-standardized health care use is treated as the measure of horizontal inequity. Need variables refer to the factors that are likely to affect individuals' health care need, such as demographic (e.g., gender, age), subjective health status (e.g., self-assessed health status), and morbidity (e.g., chronic illnesses, activity limitation) variables [24]. Non-need variables such as education level, income, and health insurance coverage are also controlled for in regression for indirect need standardization. This is not to standardize but to reduce potential bias that may arise if non-need variables correlated to need variables are omitted from the regression [21,24].

To compute a horizontal inequity index using the second method (*C*_*U *_- *C*_*N*_), the concentration index for need-predicted health care use should be estimated first [25]. The concentration index of actual health care use (*C*_*U*_) is calculated based on the observed number of health care use (e.g., number of doctor visits).

Levels of income-related inequality and horizontal inequity in health care use may differ between rural and non-rural areas due to the vast differences in demographic composition, residents' health status, income distribution, and health care use patterns and resources in the U.S. [16]. Compared to non-rural residents, rural counterparts are more likely to be older, white, and less educated, to have lower household income and more activity limitations, and to report poorer health [16,26]. Rural areas tend to have fewer primary health care providers and specialty doctors [16,27]. Rural residents are likely to have more barriers to health care [28], fewer visits to health care providers [29,30], more visits to other types of health professionals (i.e., non-physicians) [26], and fewer visits to specialty doctors [30] than their non-rural counterparts. Income inequality and its association with health also vary between rural and non-rural areas. Studies show that in rural areas income inequality measured by Gini coefficient is higher [16], and the negative impact of income inequality on health outcomes is greater than in non-rural areas [16,31]. Given these geographic differences, it is crucial to examine income-related inequality and horizontal inequity in health care use by rural-urban distinction in order to better understand health inequalities and inequities in the U.S. [32].

This study makes an important contribution to the literature in several ways. First, this study not only quantifies the magnitude of income-related inequalities in health care use but also measures the extent to which health care use is related to income after differences in health care need are standardized. Second, we measure the potential influences of known determinants of health care use on income-related inequalities by decomposing the inequalities into contributions of need- and non-need-related factors. Third, we not only measure the overall horizontal inequity but also compare the degrees of horizontal inequities between subgroups. Fourth, this study also examines whether income-related inequalities, determinants' of inequalities, and horizontal inequities differ between rural and non-rural settings. This study aims to: 1) decompose income-related inequalities in health care use by source of the inequalities among U.S. non-elderly population; 2) measure degrees of horizontal inequities adjusting for differences in health care need across the income distribution; and 3) compare income-related inequalities, determinants of inequalities, and horizontal inequities by rural-urban locale.

## Methods

### Data source

This analysis used pooled data of non-elderly adult samples aged 18 to 64 from the 1998, 1999, 2000, and 2001 U.S. National Health Interview Survey (NHIS). NHIS is an annual survey of a nationally representative household sample using random-digit dialed telephone interviews to collect information on a broad range of health topics including illness, disability, chronic impairments, and health care use of noninstitutionalized civilian population of the 50 states and the District of Columbia [33]. The area of residence was categorized as either rural if it belonged to non-Metropolitan Statistical Area (MSA) or non-rural if it belonged to MSA according to the definition of the U.S. Office of Management and Budget [34].

### Variables

The dependent variable is the frequency of ambulatory health care use as measured by the number of health professional contacts including visits to doctors' office, telephone contacts, and home care services during the past two weeks. Health professional refers to physicians, nurses, and other health care professionals such as physical therapists, ophthalmologists, and chiropractors [33].

Equivalent individual income was calculated based on reported family income using the modified Organization for Economic Co-operation and Development (OECD) equation formula. Equivalent individual income is defined as the income of an individual adjusted for differences in family size and composition. The equivalence scale formula is:

Family income included all sources (wages, salaries, and all transfers including non-taxable transfers) from all family members who were 18 years or older.

In computing the concentration index of need-predicted use (*C*_*N*_), the contribution of the following need variables was included: 6 categories of age-sex variables (5 dummy variables with a reference group of 18-24 year-old female adults), 4 dummy variables derived from a five-point Likert-type scale of self-assessed health status (excellent health as a reference), and dummy variables for activity limitations and illnesses. Activity limitation was coded 1 if a respondent reported any activity limitation due to mental, physical, or emotional problems and 0 otherwise. Illness was also coded 1 if a respondent reported any illness condition and 0 otherwise.

In addition to need variables, we included several non-need variables that are known to be correlated with both health care use and income [24] in need standardization and decomposition analyses. These non-need variables include race (a dummy variable for non-white), education level (dummy variables for below-high-school and above-high-school education), health insurance status (dummy variables for the uninsured and the publicly insured), and usual source of care (a dummy variable for no usual source of care).

### Statistical analysis

In statistical analyses, steps were taken to correct errors occurring in pooled data with different sampling weights by year. Fahimi's weighting method for pooled data [35] was used to adjust for different sampling weights of multiple-year surveys. The suggested method for composite weight (*w_ki_*) is as follows:(1)

where N is the population size, *k*, is the number of surveys to be pooled, *W_ki _*is the sampling weight for the *i *th sample of *k *survey datasets. The sample size of pooled data is *n *= *n*_1 _+ *n*_2 _+ ......... + *n_k_*. To avoid inflated variance in pooled data, weights (*W_ki_*) from each sample have to be normalized with respect to their corresponding sample sizes [35]. For precise statistical inference of clustered and serially correlated data, robust standard errors were computed using the Huber-White sandwich estimator of variance [36] using the cluster and robust option in Stata [37].

A concentration index measuring income-related inequalities in health care use was calculated using a convenient regression method as follows:(2)

where  is the variance of r_i_, and *μ *is the mean of health care use y_i_, and r_i _is the weighted fractional rank of utilized income, defined as(3)

where w_i _is the sample weight scaled to sum to 1 [24]. The concentration index (C) of health care use is equal to  estimated from Equation (2).

The concentration index was decomposed into the contribution of individual factors to income-related inequalities following the method proposed by van Doorslaer, Koolman, and Jones [21]. For nonlinear models, decomposition is possible only if some linear approximation is made [21]. Linear approximation is expressed as:(4)

where *α*^m ^is an intercept, *ε_i _*is an error term, and *δ*^m^, *β*^m^_*k *_and *γ*^m^_*j *_are the partial effect of log income (*ln inc*), a set of *k *need and j non-need variables, respectively. Given the relationship between *y*_*i *_and *x*_*ki*_, the overall inequalities in health care use *C *is expressed as(5)

where *μ *is the mean of *y*, *C_k _*and *C_j _*are the concentration index of *x_k _*and *z_j_*, and *GC_ε _*is the generalized concentration index of the error tem *ε*. The overall inequality in an outcome has two components: an explained component and an unexplained component. The residual component reflects the income-related inequality that is not explained by systematic variation of the determinants in the model. The explained component has two main elements: the impact of each determinant on an outcome and the extent of unequal distribution of each determinant across income groups [38].

The extent of horizontal inequity was measured by the concentration index of need-standardized health care use and by subtracting the concentration index of need-predicted use (*C*_*N*_) from that of actual health care use (*C*_*U*_) as follows [39]:

where *HI*_*wv *_is the horizontal inequity index, *C*_*U *_is the concentration index of health care use before need standardization, and *C*_*N *_is the need-predicted concentration index (the contribution of need variables to the total inequality). Horizontal inequity indices for subgroups (by race, education level, insurance type, and usual source of care) were also calculated to examine whether the degree of horizontal inequity varies across the subgroups. In calculating horizontal inequity indices for subgroups, all independent variables except for the group variable of interest were included in need standardization. For example, in measuring the horizontal inequity indices of white and non-white subgroups, all independent (need and non-need) variables were included in need standardization except for the race indicator variable. All analyses were performed with the whole sample and with rural and non-rural samples separately.

## Results

### Sample characteristics

Table 1 displays sample characteristics and means and concentration indices of ambulatory care use when weights are applied. Compared to non-rural residents, rural residents were more likely to be white and older and to report lower socioeconomic status, poorer health, and unfavorable health insurance coverage. Rural residents were less likely to assess their health as excellent or very good (64% vs. 71%), and more likely to have chronic illnesses (28% vs. 22%) and activity limitations (14% vs. 10%) than non-rural counterparts. Approximately 20% of rural residents were uninsured, compared to 16% of adults in non-rural areas. The proportion of private insurance coverage was 6% lower in rural areas than in non-rural areas. Residents of non-rural areas used ambulatory care more often (0.386) than those in rural areas (0.377) in the past two weeks.

**Table 1:** Sample characteristics (%) and means and concentration indices of two-week ambulatory care use

	Total (n = 83,152)	Rural (n = 16,297)	Non-rural (n = 66,855)
Age			
18-24	15.54	16.66	15.24
25-44	50.29	46.88	51.21
45-64	34.17	36.45	33.55
Sex			
Female	50.43	50.99	50.28
Male	49.57	49.01	49.72
Race			
White	79.37	87.18	77.27
Non-white	20.63	12.82	22.73
Limitations*			
Yes	10.76	14.34	9.79
No	89.24	85.66	90.21
Illness			
Yes	23.61	28.41	22.33
No	76.39	71.59	77.67
Self-assessed health status			
Excellent	35.92	31.39	37.14
Very good	33.47	32.57	33.71
Good	21.98	24.49	21.31
Fair	6.57	8.42	6.08
Poor	2.06	3.13	1.77
Education			
Below high	10.12	12.61	9.45
High	31.04	37.71	29.24
Above high	58.85	49.68	61.31
Insurance			
Uninsured	17.00	19.50	16.33
Private insured	75.50	70.56	76.83
Public insured	7.50	9.94	6.84
Usual Care Source			
Yes	87.36	88.47	87.06
No	12.64	11.53	12.94
No. of two-week ambulatory care use	0.384	0.377	0.386
Crude CI (*C*_*U*_)	- 0.037	- 0.058	- 0.020
Need-predicted CI (C_*N*_)	- 0.155	- 0.107	- 0.117
*HI*_*wv *_*(C_U _- C_N_)*	0.118	0.049	0.097

CI = concentration index: HI_wv _= horizontal inequity index

*Limitations: any limitations of activity due to physical, mental, or emotional problems

Ambulatory care use was disproportionately concentrated in the poor before need adjustment. The crude concentration indices for the whole sample and rural and non-rural subsamples were negative, indicating income-related inequalities in health care use favoring the poor when health care need was ignored. The negative values of need-predicted concentration indices (-0.107 for rural and -0.117 for non-rural) signify that expected health care use given health care need is larger among the poor than among the better-off. Overall, the distribution of health care use given health care need was in favor of the better-off as indicated by the positive value of the horizontal inequity index (0.118). Each rural and non-rural population also showed pro-rich horizontal inequity in ambulatory care use.

### Decomposition analyses

Decomposition analyses illustrate the sources of pro-poor income-related inequalities in ambulatory care use. Table 2 presents the results of decomposition analyses with need variables (age, gender, activity limitation, illness, and self-assessed health) and non-need variables (race, education, income, health insurance status, and usual source of care) for each rural and non-rural population. Regression coefficients represent the partial effect of each determinant on the outcome (number of ambulatory care use) conditional on other covariates in the model. The reference group in the model was 18-24 year-old white female adults who had high school education, average income, private health insurance, and usual source of care without activity limitation and illness, and assessed one's health as excellent.

**Table 2:** Decomposition analysis of factors contributing to income-related inequalities in ambulatory care use

	Rural			Non-rural		
	
	Regression coefficient (95% CI)	Concentration index	Contribution %*	Regression coefficient (95% CI)	Concentration index	Contribution %*
**Demographic characteristics**						
Female						
25-44	-0.046 (-0.075,-0.018)	-0.002	-0.071	0.020 (-0.042,0.081)	-0.019	0.778
45-64	-0.041 (-0.064,-0.017)	0.069	2.077	0.030 (-0.065,0.126)	0.077	-3.259
Male						
18-24	-0.163 (-0.220,-0.106)	-0.269	-14.621	-0.169 (-0.273,-0.066)	-0.275	-29.050
25-44	-0.150 (-0.160,-0.140)	0.050	6.833	-0.149 (-0.195,-0.104)	0.040	12.562
45-64	-0.137 (-0.186,-0.088)	0.140	13.791	-0.108 (-0.203,-0.012)	0.163	23.948
Race						
Non-white	-0.027 (-0.056,0.002)	-0.322	-4.466	-0.046 (-0.047,-0.045)	-0.224	-19.233
						
**Socioeconomic characteristics**						
Education						
Below high	-0.045 (-0.068,-0.021)	-0.371	-8.349	-0.072 (-0.104,-0.040)	-0.512	-28.394
Above high	0.088 (0.064,0.112)	0.140	-24.644	0.074 (0.037,0.111)	0.148	-55.095
Log of equivalent income	0.019 (0.000,0.038)	0.040	-30.306	0.016 (0.008,0.024)	0.040	-52.419
						
**Healthcare system factors**						
Insurance						
Uninsured	-0.059 (-0.087,-0.031)	-0.360	-16.620	-0.132 (-0.155,-0.108)	-0.468	-82.305
Public-insured	0.060 (0.029,0.092)	-0.407	9.757	0.036 (0.028,0.043)	-0.536	10.738
Usual source of care						
No	-0.143 (-0.159,-0.126)	-0.193	-12.734	-0.136 (-0.200,-0.071)	-0.218	-31.365
						
**Health-related factors**						
Limitations**						
Yes	0.355 (0.191,0.519)	-0.282	57.551	0.684 (0.549,0.819)	-0.277	152.279
Illness						
Yes	0.144 (0.111,0.178)	-0.112	18.438	0.150 (0.090,0.209)	-0.078	21.262
SAH						
Good	0.069 (0.056,0.082)	0.053	-4.801	0.108 (0.057,0.160)	0.038	-11.361
Fair	0.153 (0.123,0.184)	-0.072	10.827	0.234 (0.120,0.347)	-0.117	47.780
Poor	0.304 (0.259,0.349)	-0.307	31.492	0.569 (0.358,0.781)	-0.344	97.439
Very poor	0.614 (0.372,0.857)	-0.513	39.630	1.273 (1.040,1.505)	-0.507	93.495

SAH = self-assessed health status: CI = confidence interval

*Unadjusted contribution percentage

**Limitations: any limitations of activity due to physical, mental, or emotional problems

The associations between determinants and the outcome were in expected directions. Having health-related problems (activity limitation and illness), perceiving one's health not excellent, and having higher education, higher income, and public insurance were associated with more ambulatory care use when all other factors were equal. Being non-white or less educated and having no insurance or usual source of care were associated with less ambulatory care use in both rural and non-rural populations when all other factors were equal.

Decomposition of concentration indices also showed expected distribution of determinants in income groups. Positive (negative) concentration indices indicate concentration of individuals with specific characteristics in higher (lower) income groups. For example, individuals with above-high-school education were concentrated in higher-income groups (concentration index 0.140 for rural and 0.148 for non-rural). In contrast, individuals with below-high-school education were disproportionately concentrated in lower-income groups (concentration index -0.371 for rural and -0.512 for non-rural). Non-whites, people with public insurance, people without health insurance or a usual source of care, and those in poor health were disproportionately concentrated in low-income groups.

The last column for each locale in Table 2 lists the unadjusted percentage contribution of each determinant to the total observed income-related inequalities in ambulatory care use. A positive (negative) value of the contribution indicates that the total inequalities in health care use favoring the poor, with other things equal, would be lower (higher) if the determinant was equally distributed across income groups or if the determinant was not associated with the health care use outcome (instead of being associated with the outcome as indicated by the coefficient in the decomposition model). For almost all determinants, the direction of the contribution was the same across locales: if a determinant had a negative contribution to the pro-poor distribution in rural population, it had also a negative contribution in non-rural population.

For both rural and non-rural populations, health-related variables (ill health) as a group were the largest contributor to income-related inequalities because of their unequal distribution in low-income groups and strong positive association with health care use. Activity limitation was the single largest contributor to income-related inequalities as indicated by 57% in rural and 152% in non-rural areas. It can be interpreted that the total inequalities in health care use favoring the poor, with all else equal, would have been 57% lower in rural areas if individuals with activity limitations were equally distributed across income groups or if having activity limitation was not associated with health care use as indicated by the regression coefficient (0.355) in the model. Both SES variables (education levels and income) showed negative inequality contributions. For example, due to their concentration in low-income (high-income) groups and negative (positive) association with health care use, both education groups (below-high and above-high school groups) lowered the pro-poor income-related inequalities. The negative inequality contribution of being uninsured was particularly strong in non-rural areas. In other words, the higher concentration of the uninsured in low-income groups and their lower number of ambulatory care use substantially decreased the extent of pro-poor income-related inequalities in health care use in non-rural areas.

### Horizontal inequity

The results of horizontal inequity analyses indicate that the disproportionate concentration of health care use in low-income groups was due to their greater health care need. When need was accounted for, ambulatory care use was actually distributed favoring high-income groups.

Horizontal inequity indices as measured by the concentration index of the need-standardized health care use (0.076 for rural; 0.108 for non-rural) were similar, but not identical to the concentration indices computed by subtracting *C*_*N *_from *C*_*U *_(0.049 for rural; 0.097 for non-rural) (Table 1). The positive values of the horizontal inequity indices indicate pro-rich horizontal inequity in ambulatory care use across locales. The smaller horizontal inequity in rural areas signifies relatively more equitable health care use within the rural population compared to the distribution in non-rural areas. This difference appears to be due to the relatively wider gap between health care need and actual use in non-rural areas than that in rural areas.

Horizontal inequity can also be assessed based on the regression coefficients from decomposition analyses (Table 2). Assuming that only need variables (age, gender, and health-related factors) should affect health care use, the coefficients of non-need variables (race, SES, and health care system factors) should be zero if there is no horizontal inequity with regard to non-need factors [40]. Therefore, the non-zero (positive or negative) coefficients of non-need variables in Table 2 suggest the presence of horizontal inequity with respect to these variables.

Table 3 presents variation in horizontal inequities by subgroup. Racial minorities, high-school graduates, and those with private health insurance, and people lacking a usual source of care were likely to experience relatively higher degrees of horizontal inequities with some geographic variation. Although significant pro-rich horizontal inequities were observed in most subgroup analyses of non-rural residents, in rural areas, horizontal inequities were statistically significant only among those with private health insurance and those without a usual source of care.

**Table 3:** Horizontal inequity indices and means of two-week ambulatory care use by subgroup

	Rural		Non-rural	
***HI*_*wv*_**	**Index (95% CI)**	M*	**Index (95% CI)**	M*
				
		[m**]		[m**]

White	0.034	0.378	0.065	0.340
	(-0.001, 0.070)	[0.383]	(0.046, 0.083)	[0.405]
Non-white	0.057	0.365	0.089	0.340
	(-0.014, 0.128)	[0.398]	(0.057, 0.121)	[0.347]
Below high school	0.013	0.384	-0.001	0.320
	(-0.061, 0.088)	[0.395]	(-0.045, 0.043)	[0.341]
High school	-0.006	0.337	0.055	0.346
	(-0.064, 0.052)	[0.349]	(0.023, 0.087)	[0.342]
Above high school	0.001	0.404	0.024	0.416
	(-0.042, 0.043)	[0.411]	(0.004, 0.044)	[0.424]
Uninsured	0.029	0.239	0.050	0.194
	(-0.045, 0.104)	[0.250]	(-0.001, 0.101)	[0.203]
Private	0.042	0.336	0.042	0.375
	(0.005, 0.078)	[0.338]	(0.026, 0.059)	[0.374]
Public	0.017	0.935	0.032	0.974
	(-0.039, 0.072)	[0.972]	(0.002, 0.063)	[0.989]
Usual source of care	0.032	0.411	0.074	0.422
	(-0.001, 0.066)	[0.419]	(0.057, 0.090)	[0.423]
No usual source of care	0.123	0.109	0.151	0.147
	(0.008, 0.238)	[0.119]	(0.084, 0.218)	[0.146]

CI = confidence interval: HI_wv _= horizontal inequity index

*M and m** refer to the mean of two-week ambulatory care use and its need-standardized share estimated using a zero-inflated negative binomial model, respectively.

Horizontal inequity in health care use was present within both whites and non-whites in non-rural areas, but was more substantial among non-whites (*HI*_*wv *_= 0.089 vs. 0.065). Education seems to significantly reduce horizontal inequities in non-rural areas, where the high-school graduate group had greater horizontal inequity (*HI*_*wv *_= 0.055) than the group with higher education (*HI*_*wv *_= 0.024). In rural areas, examination of horizontal inequity by insurance coverage showed pro-rich horizontal inequities only among residents with private health insurance. In non-rural areas, horizontal inequities were significant among both privately and publicly insured groups. Across locales, having a usual source of care or not appears to be closely linked to the extent of horizontal inequity. Rural residents without a usual source of care experienced significant horizontal inequity (*HI*_*wv *_= 0.123). In non-rural areas, the pro-rich horizontal inequity was much greater among residents without a usual source of care (*HI*_*wv *_= 0.151) than among those with (*HI*_*wv *_= 0.074).

## Discussion

This study decomposed income-related inequalities in ambulatory care use by source of inequalities and compare degrees of horizontal inequities between subgroups of rural and non-rural U.S. nonelderly population. When health care need was accounted for, ambulatory care use was disproportionately concentrated in high-income groups. Horizontal inequity was greater within non-rural areas than in rural areas with subgroup variation.

Regardless of locale, ambulatory care use was disproportionately concentrated in low-income groups when health care need was not considered. However, decomposition analyses demonstrated that the pro-poor distribution was in large part explained by the unequal concentration of people with greater health care need in low-income groups. Policy-relevant factors also contributed to income-related inequalities in health care use. Consistent with the findings of existing studies [12,17,40], the unequal concentration of individuals with disadvantageous characteristics (non-white (in non-rural areas), low education level, uninsured, and lacking a usual source of care) in low-income groups and their lower levels of health care use affected income-related inequalities.

Horizontal inequity indices indicate the reversion of health care use favoring the poor to favoring the better-off once health care need was adjusted. This result is in accordance with the findings of previous studies of the U.S. [41,42] and European countries [21,40,42,43]. In a study using the U.S. 1996-1998 Medical Expenditure Panel Survey [41], adjusting for health care need, predicted health care expenditures per person rose with an increase in income, indicating pro-rich horizontal inequity. A study on horizontal inequities in physician and dental care use among 50 or older adults [42] showed clear pro-rich horizontal inequities in the U.S. In England, even with the National Health Service system, there was inequity in health care use during the 1998-2000 period with respect to income, ethnicity, employment status, and education before the National Health Service reforms, which were intended to decrease inequity [40]. Studies of OECD member countries showed overall pro-rich horizontal inequities in doctor visits with weak evidence on general practitioner visits but strong evidence on specialist and dentist visits [21,43].

Subgroup comparisons demonstrated variation in horizontal inequities in this NHIS sample. Since neither the subgroup differences in degrees of horizontal inequity nor the reasons for the differences have been reported in existing studies, we can only describe the variation and speculate possible reasons. Caution should be taken in interpreting horizontal inequities of subgroups. A smaller horizontal inequity index for a subgroup does not necessarily mean that health care environment is better for the subgroup or health care is more frequently used by the group compared to another subgroup with a larger horizontal inequity index. A horizontal inequity index for a subgroup only represents how health care use is distributed in relation to income for equal need within the subgroup.

Horizontal inequity was larger among non-whites than whites, but it was not statistically significant in rural areas. In non-rural areas, both whites and non-whites showed significant horizontal inequities within each subgroup, but to a greater degree among non-whites. There have been numerous studies demonstrating a significant gap in health care use between whites and non-whites and among rural non-white subgroups in the U.S. [44-47]. However, between-group differences in horizontal inequity by race or the reasons for the differences are unknown. Yet, considering the vast heterogeneity within the group designated as non-white and potential large income inequalities within this diverse group, it is not surprising to observe greater horizontal inequity. Significant and relatively large horizontal inequities were found in other subgroups including those with high school education, with private insurance, and without a usual source of care. These horizontal inequities are also likely due to the large within-group variation in need and non-need related characteristics and the unequal health care use patterns associated with the characteristics.

There are possible limitations to be considered in interpreting our findings. First, the indices cannot provide adequate explanations for disparities that may arise from quality of care. A study reports that inequality indices were close to those measured using the average relationship between need and treatment for the population as a whole when differences in treatments between patients were ignored [48]. Therefore, inequality patterns found in our analyses are likely to underestimate the true underlying inequality since health care quality was not accounted for and the quality of care our sample received may have not been the same for different income groups. Omitted variables are another concern. Variables used for standardizing need in our analyses may not fully assess respondents' latent health status and health care need. Information on illness severity, provider characteristics, and individual preference might improve the assessment. Last, this study does not depict the whole and precise picture of income-related inequalities and horizontal inequities in health care use. Our analyses explored only ambulatory health care contacts. It did not include hospitalization. This study did not differentiate health care types such as specialty care and other health professional visits either. All ambulatory health care contacts regardless of the types of health care professionals were included in the count of ambulatory care use.

Despite these limitations, our study has unique strengths that help add to the literature. First, our analyses measured the extent to which health care use was related to income after differences in health care need were standardized beyond quantifying income-related inequalities. Second, we measured the contributions of need and non-need related determinants to income-related inequalities using decomposition analyses. Third, we compared the extent of horizontal inequities between subgroups. Fourth, this study examined differences in income-related inequalities, determinants' of the inequalities, and horizontal inequities between rural and non-rural settings.

## Conclusions

Decomposition analyses show that pro-poor income-related inequalities in ambulatory care use before need standardization was largely due to greater health care need among low-income groups. Non-need factors including health insurance, usual source of care, and socioeconomic characteristics were also significant contributors to income-related inequalities. Having public insurance increased pro-poor income-related inequalities, while having below- and above-high-school education (as compared to high school education) and higher income and not having a usual source of care decreased the pro-poor income-related inequalities because of these determinants' associations with health care use and concentration in the income distribution. Horizontal inequity indices indicate health care use favoring the better-off in both rural and non-rural areas when health care need was accounted for. However, the magnitude of horizontal inequity was greater in non-rural areas. Racial minorities, high school graduates, those with private health insurance, and people lacking a usual source of care were likely to experience relatively higher degrees of within-group horizontal inequities than their respective counterparts with some geographic variation.

The findings of this study provide information for policy makers to reduce inequalities in health care use and, in turn, health disparities. Different contributions of determinants to income-related inequalities in health care use and variation in horizontal inequities by subgroup and locale point to more explicit targets for policy amendment and resource allocation for each rural and non-rural area.

## Competing interests

The authors declare that they have no competing interests.

## Authors' contributions

HS analyzed data, interpreted the results, and drafted the initial version of the manuscript. JK interpreted the results and wrote the final version of the manuscript. All authors read and approved the final manuscript.
